# Multi-Gene Next-Generation Sequencing Panel for Analysis of *BRCA1*/*BRCA2* and Homologous Recombination Repair Genes Alterations Metastatic Castration-Resistant Prostate Cancer

**DOI:** 10.3390/ijms24108940

**Published:** 2023-05-18

**Authors:** Thais Maloberti, Antonio De Leo, Sara Coluccelli, Viviana Sanza, Elisa Gruppioni, Annalisa Altimari, Stefano Zagnoni, Francesca Giunchi, Francesco Vasuri, Michelangelo Fiorentino, Veronica Mollica, Simona Ferrari, Sara Miccoli, Michela Visani, Daniela Turchetti, Francesco Massari, Giovanni Tallini, Dario de Biase

**Affiliations:** 1 Solid Tumor Molecular Pathology Laboratory, IRCCS Azienda Ospedaliero-Universitaria di Bologna, 40138 Bologna, Italy; thais.maloberti2@unibo.it (T.M.); antonio.deleo@unibo.it (A.D.L.); viviana.sanza@ausl.bo.it (V.S.); elisa.gruppioni@aosp.bo.it (E.G.); annalisa.altimari@aosp.bo.it (A.A.); stefano.zagnoni@aosp.bo.it (S.Z.); giovanni.tallini@ausl.bo.it (G.T.); 2 Department of Medical and Surgical Sciences (DIMEC), University of Bologna, 40138 Bologna, Italy; michelangelo.fiorentino@unibo.it (M.F.); daniela.turchetti@unibo.it (D.T.); 3 Pathology Unit, IRCCS Azienda Ospedaliero-Universitaria di Bologna, 40138 Bologna, Italy; francesca.giunchi@aosp.bo.it (F.G.); francesco.vasuri@aosp.bo.it (F.V.); 4 Pathology Unit, Maggiore Hospital, AUSL Bologna, 40133 Bologna, Italy; 5 Medical Oncology, IRCCS Azienda Ospedaliero-Universitaria di Bologna, 40138 Bologna, Italy; veronica.mollica7@gmail.com; 6 Unit of Medical Genetics, IRCCS Azienda Ospedaliero-Universitaria di Bologna, 40138 Bologna, Italy; simona.ferrari@aosp.bo.it (S.F.); sara.miccoli@studio.unibo.it (S.M.); 7 Department of Pharmacy and Biotechnology, University of Bologna, 40126 Bologna, Italy; michela.visani@unibo.it

**Keywords:** prostatic adenocarcinoma, mCRPC, mutations, next-generation sequencing, BRCA1, BRCA2, HRR

## Abstract

Despite significant therapeutic advances, metastatic CRPC (mCRPC) remains a lethal disease. Mutations in homologous recombination repair (HRR) genes are frequent in mCRPC, and tumors harboring these mutations are known to be sensitive to PARP inhibitors. The aim of this study was to verify the technical effectiveness of this panel in the analysis of mCRPC, the frequency and type of mutations in the *BRCA1*/*BRCA2* genes, as well as in the homologous recombination repair (HRR) genes. A total of 50 mCRPC cases were analyzed using a multi-gene next-generation sequencing panel evaluating a total of 1360 amplicons in 24 HRR genes. Of the 50 cases, 23 specimens (46.0%) had an mCRPC harboring a pathogenic variant or a variant of uncertain significance (VUS), whereas in 27 mCRPCs (54.0%), no mutations were detected (wild-type tumors). *BRCA2* was the most commonly mutated gene (14.0% of samples), followed by *ATM* (12.0%), and *BRCA1* (6.0%). In conclusion, we have set up an NGS multi-gene panel that is capable of analyzing *BRCA1*/*BRCA2* and HRR alterations in mCRPC. Moreover, our clinical algorithm is currently being used in clinical practice for the management of patients with mCRPC.

## 1. Introduction

Prostatic cancer is the most common male neoplasia in Europe, the Americas, Australia, and Africa, with a total of 19,292,789 new cases worldwide and a world mortality rate in 2020 equal to 7.7 per 100,000 [1].

Among the mutations that convey a modest increase in risk, there are several DNA repair genes, including loss-of-function mutations in *BRCA2* (required for repair by homologous recombination) and in DNA mismatch repair genes. *BRCA1* mutations have also been associated with increased prostate cancer risk, although with less magnitude of risk [2]. Mutations in DNA mismatch repair genes associated with Lynch syndrome (*MLH1*, *MSH2*, *PMS2*, *MSH6*, and *EPCAM*) have been associated with a modest increase in prostate cancer risk, particularly for *MSH2* [2,3]. Additional genes on prostate cancer panels confer variable risks for prostate cancer, such as, for example, *CHEK2* which has been reported to confer a modest increase in risk for prostate cancer [2]. *ATM* and *PALB2* have limited data for prostate cancer risk but may be important when considering precision treatment, such as PARP inhibitors in the metastatic setting.

From a clinical point of view, prostate cancer is a particularly complex neoplasia; the pharmacological treatment, in fact, varies according to the characteristics of the tumor, such as the size, site, and degree of aggressiveness. Similar to other types of tumors, this too can be well localized at the level of the prostate gland or, in the most serious cases, present metastases, especially present in the case of castration-resistant prostate cancer, with a consequent lower survival [4]. Due to the presence of bone metastases at the time of diagnosis in the majority of patients, the possibility of treatment with surgery and/or radiotherapy is limited to a small percentage of cases, whereby hormone therapy or chemotherapy is normally used. In some cases, there is the progression of the disease despite androgen deprivation therapy (ADT). In this case, the tumor is defined as castrate-resistant prostate cancer (CRPC) or castration-resistant prostate cancer [5].

Mutations in homologous recombination repair (HRR) genes are frequent in advanced prostate cancer, and tumors harboring these mutations have known sensitivity to PARP inhibitors. The mutations in HRR genes, commonly investigated in metastatic castrate-resistant prostate cancer (mCRPC), including *BRCA1*, *BRCA2*, *ATM*, *ATR*, *BRIP1*, *CDK12*, *CHEK1*, *CHEK2*, *DSS1*, *FANCA*, *FANCD2*, *NBSI*, *PALB2*, *RAD51B*, *RAD51C*, *RAD51D*, *RAD54*, and *RPA1* [6]. The presence of pathogenic HRR mutations has been associated with early onset of disease, aggressive tumors, higher recurrence, and poor prognosis [7,8,9,10]. 

Despite significant therapeutic advances, mCRPC remains a lethal disease. The identification of specific novel predictive biomarker mutations in mCRPC is opening up new therapeutic targets. In this context, mutations in genes involved in DNA damage repair (DDR) through the homologous recombination repair (HRR) pathway have been identified in 15% to 25% of mCRPC cases [6,7].

PARP plays a role in mediating the repair of DNA single-strand breaks [11]. Olaparib, a poly (adenosine diphosphate-ribose) polymerase (PARP) inhibitor (PARPi) used in the treatment of several neoplasms [12,13,14,15,16,17], traps PARP on DNA, leading to double-strand breaks in cells undergoing DNA replication. In normal cells, the recombination repair (HRR) system repairs these breaks; however, in HRR-deficient cells, failure to repair these breaks results in cell death [18,19,20].

Olaparib has been approved for the treatment of patients with deleterious or suspected deleterious germline or somatic HRR gene-mutated mCRPC, whereas rucaparib has been approved for those with deleterious *BRCA1* or *BRCA2* (germline and/or somatic) mutation-associated mCRPC in patients who have progressed (following prior treatment with enzalutamide or abiraterone) [21].

Upon PARPi approval, the National Comprehensive Cancer Network (NCCN) updated guidelines (version two, 2020) now recommend germline and/or somatic HRR gene panel and *BRCA1*/*BRCA2* testing to identify pathogenic mutations for treatment with olaparib and rucaparib [22]. 

Olaparib received approval based on the phase three PROfound trial (ClinicalTrials.gov identifier NCT02987543) [16], demonstrating improved outcomes in patients with mCRPC who had failed prior androgen receptor-directed therapy and had homologous recombination repair gene mutations in *BRCA1*, *BRCA2*, or *ATM* (cohort A) or *BRIP1*, *BARD1*, *CDK12*, *CHEK1*, *CHEK2*, *FANCL*, *PALB2*, *PPP2R2A*, *RAD51B*, *RAD51C*, *RAD51D*, or *RAD54L* (cohort B) [16]. Rucaparib received approval based on the phase two TRITON2 trial (ClinicalTrials.gov identifier NCT02952534) in patients with BRCA-mutant mCRPC [23]. On the basis of the interim results from the phase two GALAHAD trial (ClinicalTrials.gov identifier NCT02854436), niraparib received breakthrough therapy designation for the treatment of BRCA-mutant mCRPC. Niraparib demonstrated clinical activity in patients with treatment-refractory mCRPC who showed durable responses, particularly in biallelic *BRCA* mutation carriers [24].

The next-generation sequencing panel can detect different genetic aberrations, point mutations, indels, and copy number variations (CNVs) in a single test. Even if commercial NGS panels are commonly available, the NGS gene panels may also be customizable and provide flexibility to select therapeutically actionable genes for specific testing purposes of germline and tissue testing [25]. For example, laboratory-developed panels may be designed for analyzing only *BRCA1*/*BRCA2* and the other HRR genes clinically relevant in prostate cancer [6].

Though a higher prevalence of HRR mutations was obtained from the metastatic tissue samples, less than 5% of metastatic samples were from bone tissue, emphasizing the inaccessibility of bone metastatic tissue [6,16]. Although a higher prevalence of mutations is observed in metastatic tissue, obtaining a sample from a metastatic site is difficult in mCRPC, as the most frequent site of metastasis is the bone. The heterogeneity of the tumor tissue itself is a restriction because it may not accurately reflect the biology of the tumor and, as a result, its overall genetic mutation profile. Improper fixation of tumor samples poses specific challenges to the integrity of DNA. Fresh-frozen samples are a feasible sample type for genetic analysis. However, in clinical settings, it may not always be possible to perform a rebiopsy, and the determination of tumor content may also be a challenge before proceeding with NGS-based HRR gene testing. In such cases, archived samples are often used, with FFPE samples being the most preferred option [26,27,28]. Tumor content evaluation of FFPE samples is critical for identifying successful genetic alterations through NGS gene panel testing. If the tumor content is inadequate, it is advisable to obtain microdissected target tissue by a trained pathologist to enrich the tumor content [29].

Given the importance of mutational analysis and PARP inhibitors in the treatment of prostate cancer, the aim of this study was the development of a next-generation sequencing panel for the molecular characterization of prostate cancer. In particular, with this study, we wanted to verify the technical effectiveness of this panel, the frequency and type of mutations in the *BRCA1*/*BRCA2* genes, as well as the possible presence of mutations or mutations in the HRR (homologous recombination repair) genes, and the significance of possible VUS (variants of uncertain significance) in mCRPC.

## 2. Results

### 2.1. NGS Panel Performance

**Figure 1:**
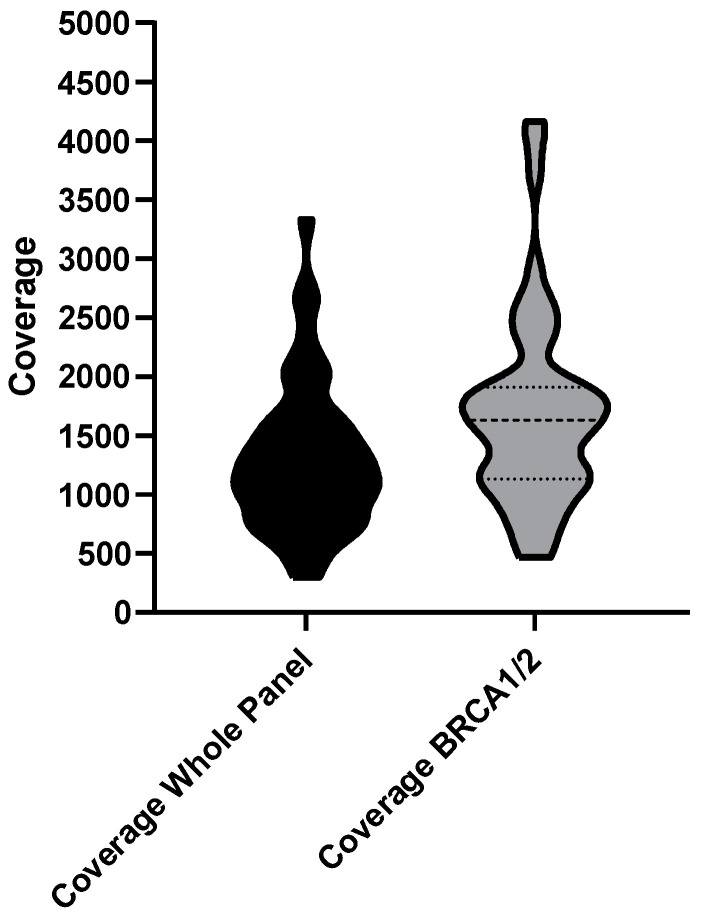
Comparison of the mean amplicon coverage in the whole panel (left) and in amplicons covering the *BRCA1*/*BRCA2* regions (right).

The mean coverage of the whole panel was 1291.5 (ranging from 297 to 3334.7), whereas the mean coverage of the amplicons covering *BRCA1*/*BRCA2* regions was 1684.6 (ranging from 468 to 4162) (Figure 1).

**Figure 2:**
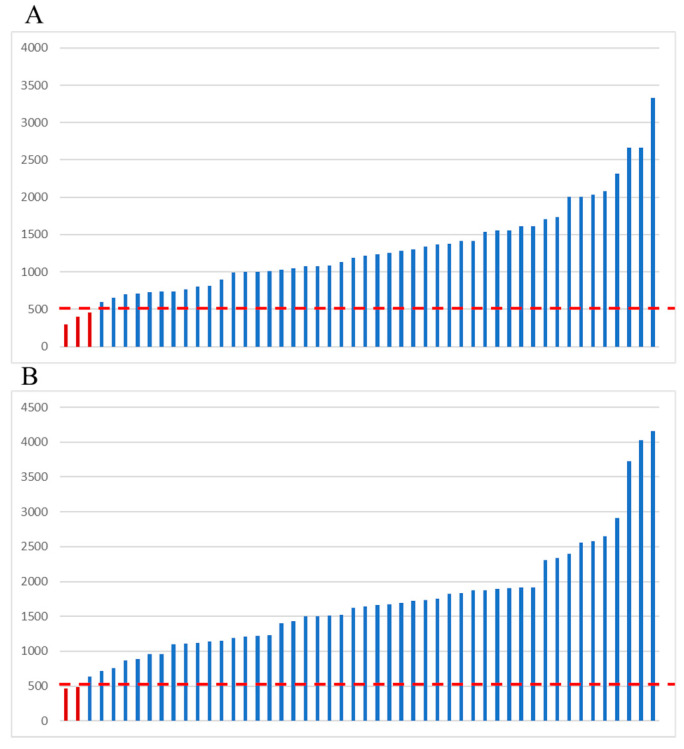
Mean coverage of the amplicons analyzed using the custom-designed panel. (**A**) Whole panel; (**B**) *BRCA1*/*BRCA2* amplicons. Dotted red line: 500× coverage. Red bars: samples with a mean amplicon coverage below 500×. *y* axis: amplicon coverage.

In three of the fifty samples (6.0%), the mean coverage of the whole panel was below 500× (ranging from 297.0 to 460.6) (Figure 2A), while in two of the fifty samples (4.0%) the mean coverage of the *BRCA1*/*BRCA2* regions was below 500× (ranging from 468.3 to 480.5) (Figure 2B).

**Figure 3:**
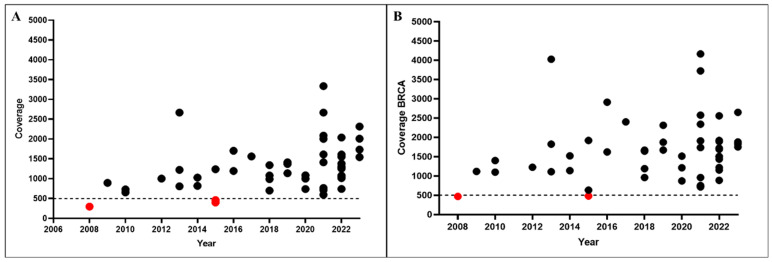
(**A**) Coverage of the whole panel in samples grouped according to the year of surgery/biopsy. (**B**) Coverage of the *BRCA1*/*BRCA2* amplicons in samples grouped according to the year of surgery/biopsy. Dotted line: 500× coverage. Red circles: samples with coverage below 500×.

Of the three samples with a mean coverage below 500×, two were biopsy specimens and one was a surgical resection, all from primary prostatic lesions. Of these three specimens, one was from a biological block dated 2008 and two were from 2015 (Figure 3). None of the samples from 2016 to 2023 had a coverage below 500× (Figure 3).

### 2.2. Mutational Analysis in Neoplastic Tissue (Somatic Alterations)

**Figure 4:**
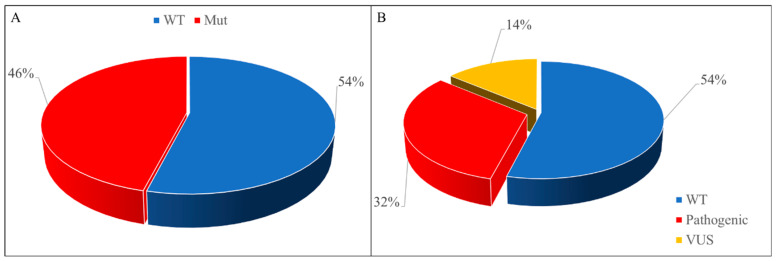
Frequency of HRR mutations in mCRPC patients. (**A**) Patients with pathogenic or VUS mutations (Mut) vs. patients without mutations (WT). (**B**) Frequency of mutations distinguishing “pathogenic mutations” from “VUS” ones.

A total of 23 patients (46.0%) harbored a pathogenic variant or a VUS, whereas in 27 samples (54.0%), no variants were detected (wild-type tumors) (Supplementary Table S1, Figure 4A). Of the twenty-three mutated samples, sixteen harbored at least one pathogenic mutation, whereas in seven, only VUS variants (but not pathogenic ones) were detected (Figure 4B). 

*BRCA2* was the most commonly mutated gene (14.0% of patients, 12.0% if only pathogenic/likely pathogenic variants were considered), followed by *ATM* (12.0%, but only 2.0% if VUS alterations were not included), and *BRCA1* (6.0%) (Table 1). Other altered genes were found in one to two patients (Table 1). In three cases (#11, #19, and #27, Supplementary Table S1) the *BRCA1*/*BRCA2* mutations were confirmed using another NGS panel (Oncomine *BRCA1*/*BRCA2*, Thermo Fisher Scientific, Waltham, MA, USA), and in all cases the mutations were confirmed. For the other *BRCA1*/*BRCA2* mutated samples, not enough material was available to perform another test.

Overall, a total of 35 pathogenic/likely pathogenic/VUS variants were detected in the HRR genes. *BRCA2* was the most commonly mutated gene, accounting for nine of the thirty-five mutations (25.7%), followed by *ATM* (six mutations, 17.1%), and *BRCA1* (three mutations, 8.6%) (Table 1, Supplementary Figure S1). In all the other genes, one or two variants were detected (Table 1, Supplementary Figure S1).

In seven cases, concomitant mutations were detected (Table 1): one case harbored concomitant *BRCA1* and *BRIP1* variants; one case harbored two concomitant *BRCA2* variants; one case harbored concomitant *BRCA2* and *CHEK2* variants; one case harbored *BRCA2* and *ATM* variants; one case harbored concomitant *BRCA2*, *ATM*, *BARD1*, and *CDK12* mutations; one case harbored concomitant *FANCA*, *PIK3CA*, and *PPP2R2A* variants; and one case harbored concomitant *ATM* and *RAD51D* variants. Of the 35 detected variants, 19 (54.3%) were pathogenic or likely pathogenic and the other 16 (45.7%) were VUS variants.

Intriguingly, after the first “round” of analysis, the total number of VUS variants was equal to 32. After revising the significance of the variants at the end of the study, sixteen variants were reclassified from VUS to “benign” or “likely benign” in the following genes: three variants in *ARID1A*, four in *ATM*, three in *BRIP1*, two in *CDK12*, two in *CHEK2*, and one in *RAD51B*.

#### Correlation between HRR Mutations and Age or the Gleason Score

**Figure 5:**
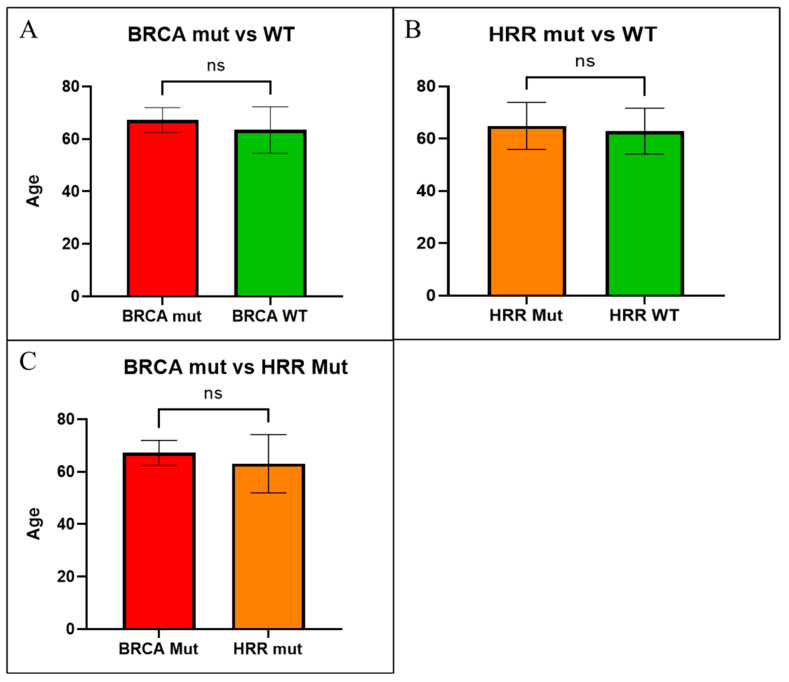
Age comparison between (**A**) *BRCA1*/*BRCA2* mutated and *BRCA1*/*BRCA2* WT tumors; (**B**) HRR mutated and HRR WT tumors; and (**C**) *BRCA1*/*BRCA2* mutated and BRCAness/HRR mutated tumors. ns = not statistically significant.

No statistically significant differences were observed in age between *BRCA1*/*2* mutated patients (mean age: 67.2 years) and *BRCA1*/*BRCA2* WT patients (mean age: 63.4 years) (*p* = 0.2451, Mann–Whitney test) (Figure 5A). Likewise, no differences in age were observed also in HRR mutated patients’ group (mean age: 64.9 years) and HRR WT patients (mean age: 62.9 years) (*p* = 0.2711, Mann–Whitney test) (Figure 5B), and between *BRCA1*/*2* mutated patients (mean age: 67.2 years) and HRR-BRCAness mutated patients (mean age: 63.1 years) (*p* = 0.4729, Mann–Whitney test) (Figure 5C). We did not find any differences when considering or excluding VUS mutations from the groups of mutated patients.

No statistically significant differences were observed in *BRCA1*/*BRCA2* mutation frequencies according to the Gleason Score (*p* = 0.6754, Chi-square test) (Table 2). Similarly, no significant differences were found in HRR gene mutations according to the Gleason Score (*p* = 0.5652, Chi-square test) (Table 2).

No statistically significant differences were observed in the frequencies of *BRCA1*/*BRCA2* (*p* = 0.5349, Chi-square test) or HRR gene mutations (*p* = 0.9221, Chi-square test) frequencies in the different Grade Score groups (Table 3).

### 2.3. Germline Analysis

In five patients, the detected variants required genetic counseling and evaluation at the germline level (Table 4). For the other detected mutations, genetic counseling was not recommended due to low VAF (<20%) or the mutated gene.

Regarding the five patients for whom genetic counseling was recommended, three have been analyzed, and two will be in the near future. Two variants, *BRCA2* c.4131_4132insTGAGGA and *MLH1* c.794G>A, were confirmed in the germline DNA. However, the latter is classified as “likely pathogenic” according to ACMG (and Tier II for AMP), but as a VUS according to ClinVar. The germline DNA from one patient harboring the *BRCA2* c.4913_4915delinsTTC and *BRCA2* c.4983T>G variants is still under evaluation.

## 3. Discussion

In a molecular analysis of 333 primary prostate cancers, the Cancer Genome Atlas (TCGA) study showed a 19% prevalence of alterations in several DNA repair genes, including *BRCA2*, *BRCA1*, *ATM*, *CDK12*, *FANCD2,* and *RAD51C* [30].

The recent PROfound study represents the largest analysis currently available on DNA repair defects in prostate cancer [16]. This phase three clinical study evaluated the efficacy of the PARP inhibitor “Olaparib” in patients with metastatic castration-resistant prostate cancer (mCRPC) and evaluated 2792 biopsies for the presence of aberrations in fifteen genes involved in DNA repair [31].

It has been demonstrated that the pathogenic variants (PV) of *BRCA1*/*BRCA2* genes, whether germline or somatic, represent a predictive biomarker of greater sensitivity to treatment with inhibitors of the enzyme poly (ADP-ribose) polymerase (PARP), which intervenes in DNA repair damaged single-strand prostate cancer in patients with hormone-resistant metastatic prostate cancer. The efficacy of PARP inhibitors as a therapeutic option in prostate cancer is achieved through a “synthetic lethality” process with a simultaneous loss of double-stranded DNA repair function by homologous recombination (HR), in which BRCA1/BRCA2 proteins play an essential role [32]. In October 2020, clinical studies led to the registration by the European Regulatory Agency EMA (European Medicines Agency) of the PARP inhibitor olaparib “*indicated, in monotherapy, for the treatment of adult patients with castration-resistant metastatic prostate cancer with BRCA1/BRCA2 gene mutations (germline PV and/or somatic PV), progressing after previous treatment including a new hormonal agent*” (https://www.ema.europa.eu/en/documents/product-information/lynparza-epar-product-information_it.pdf (accessed on 30 March 2023)). Patients must have confirmation of a PV in the *BRCA1*/*BRCA2* prostate cancer susceptibility genes (germline or somatic) before initiating treatment with olaparib. The assessment of *BRCA1*/*BRCA2* mutation status should be performed in a specialized laboratory using a validated test method [32,33,34,35]. Based on this evidence, a referral for *BRCA1*/*BRCA2* testing was proposed for men with metastatic prostate cancer.

The analysis of *BRCA1*/*BRCA2* and HRR genes is, therefore, important today both for the therapeutic management of the patient and for undertaking a possible path of oncogenic counseling in family members in order to identify high-risk carriers, to whom to propose targeted programs of early diagnosis of tumors associated with BRCA-related hereditary-familial transmission syndromes and strategies aimed at reducing the risk.

Molecular analysis of prostate samples is particularly tricky. In the PROFOUND study, FFPE tumor tissue samples were used for genetic testing. Of the 4047 samples available, the reasons for test failure in 31% of samples was pathology review failure (6.8%), DNA extraction failure (13.2%), and failure after DNA extraction (6.9%) [16].

Multiple gene targets can be tested using multi-gene NGS cancer panels. The benefits of using multi-gene panels are: (i) a large number of targets can be tested starting from the same amount of nucleic acid input (usually ~10–20 ng of DNA); (ii) costs are reduced to the minimum possible, and the price of analyzing a specific target gene is equal to the overall cost divided by the number of genes in the panel.

Moreover, the sequences of genes not initially sought by the clinician remain in lab databases. This enables quick data recovery in the event of a need (e.g., updating of recommendations or the discovery of additional predictive/prognostic indicators) without having to reextract the nucleic acid and resequence the specimen. Repeating these analyses would be difficult in samples with low amounts of biological material, such as prostate biopsies. The custom-designed multi-gene panels also have the flexibility to be quickly updated at any moment in response to the identification of unique new biomarkers or guideline revisions, irrespective of the selection and timing of commercial diagnostic tools.

In this study, we validated a laboratory-developed custom-designed multi-gene NGS panel allowing us to analyze *BRCA1*/*BRCA2* genes together with other 22 HRR and mismatch repair involved genes. Our custom-designed multi-gene NGS panel is reliable, with a relatively low overall percentage of cases with low coverage (i.e., <500×) amplicons. 

Considering that, in mCRPC, material from the metastatic site may not always be available or of poor quality/quantity (e.g., in bone metastases), the NGS panel must be successfully performed on archival prostate material. By also using our NGS panel on archived material from the primitive site, we have demonstrated that this academic panel can successfully be used in patients where metastatic specimens are unavailable, and the archived prostate sample must be used for BRCA/HRR analysis.

Our data are consistent with those reported in the literature: *BRCA1* and *BRCA2* genes were confirmed as the more frequent HRR genes altered in mCRPC (20% of analyzed samples), demonstrating that the primary prostate site can be successfully used for the analysis of *BRCA*/HRR genes in mCRPC patients. However, it should be considered that taken together, the other HRR genes were observed to be altered in 26% of mCRPCs. These data lead to the hypothesis that it could be useful to investigate other HRR genes other than *BRCA1*/*BRCA2* to obtain a more reliable molecular profile of mCRPCs. Considering that only 20% of specimens harbored *BRCA1*/*BRCA2* alterations, it would be more profitable to use a unique multi-gene panel, including *BRCA1*/*BRCA2* genes together with other HRR markers, rather than using a sequential approach, analyzing *BRCA1*/*BRCA2* as a first step and continuing with HRR analysis only in BRCAness samples. Another useful observation from the data that we have obtained is the importance of revaluing the VUS alterations. In fact, in our study, 50% of VUS variants identified have been reclassified as “benign” or “likely benign” at a subsequent evaluation (from 6 to 12 months later than the first evaluation).

The limitations of this study are due to the limited number of samples and the fact that the molecular analyses were predominantly performed on archival primary biopsies rather than metastatic samples. Even if it has been demonstrated that primary prostate tissue accurately reflects the mutational status of metastatic prostate tissue [36]. Considering that the molecular status of metastases may differ from that of the primary lesion, whenever possible (i.e., when the material is available and the nucleic acid is of good quality), it may be preferable to perform *BRCA*/HRR analysis on the metastatic specimen. However, if the metastatic sample is not available or the extracted DNA is of poor quality, as can be the case for bone biopsies, then it would be possible to perform NGS analysis on the primary lesion. Indeed, in light of the poor quality of nucleic acid obtained from bone specimens, the ESMO guidelines emphasize that the sample used for this test “should possibly not belong to bone metastasis” [37]. In the present study, it has been demonstrated that analysis of archival samples in mCRPC samples is feasible using this NGS panel, and the percentage of mutations observed in *BRCA1*/*BRCA2* and HRR genes is overlaps with that reported in the literature [8,9,38]. Another possible alternative in case of unavailable metastatic material could be to perform the test on liquid biopsy (e.g., plasma or urine [39,40]), although the application of this method is challenging due to the lack of standardized analysis methods, the need for high analytical sensitivity, and expertise in data analysis [39].

**Figure 6:**
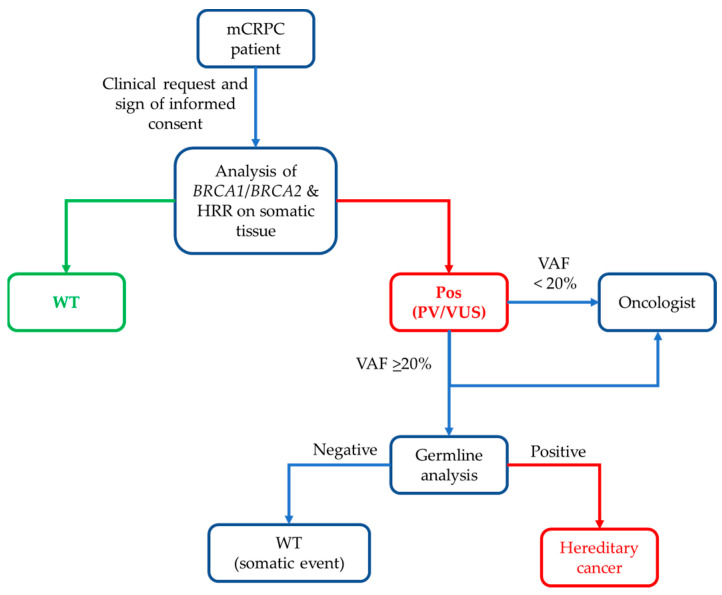
Developed algorithm for mCRPC patients’ management.

Using our analysis protocol, it was possible to create a clinical pipeline for the management of patients with mCRPC. The clinician may request the typing of *BRC1*/*BRCA2* and HRR genes on somatic tissue when necessary. The analysis is then performed on the available lesion or the most representative one. The result is then communicated to the oncologist for therapeutic management, and if a pathogenic/VUS mutation with a VAF greater than 20% is identified, the genetic counseling process is activated, thanks to the signing of the informed consent by the patient at the time of the oncological visit (Figure 6). In our series, five of fifty patients (10.0%) needed genetic counseling, three patients due to *BRCA1*/*BRCA2* mutations, and two patients due to genes connected to the mismatch repair system (*MLH1*, *PMS2*). Therefore, the algorithm is currently in use in clinical practice for the management of patients with mCRPC. Moreover, from a future perspective, the panel could also be further implemented by adding the HRD (Homologous Recombination Deficiency) evaluation, as already happens for ovarian cancers.

## 4. Materials and Methods

### 4.1. Case Selection

A total of 50 mCRPC cases were analyzed in the Molecular Laboratory of Solid Tumors, IRCCS Policlinico di S. Orsola. All cases were formalin-fixed and paraffin-embedded (FFPE), 40 cases (80.0%) were bioptic specimens, and 10 (20.0%) were surgical resections (Table 5). The age range was between 40 and 80 years old (mean 63.8 years). All 50 specimens were from metastatic CRPC. The sources of the analyzed specimens were as follows: 43 specimens (86.0%) were from primary prostatic lesions, 3 (6.0%) were from lymph nodal metastases, 2 (4.0%) were from lung metastases, 1 (2.0%) was from liver metastasis, and 1 (2.0%) was from a bladder metastatic site (Table 5). All the specimens were obtained from patients who showed disease progression after undergoing androgen deprivation therapy.

In cases where the primitive prostate site was analyzed and no mutations were found in *BRCA1*/*BRCA2* or other HRR genes (n = 26), it was not possible to perform a biopsy of the metastatic site for molecular analysis.

Of the 50 analyzed samples, 8 (16.0%) had a Gleason Score equal to 4 + 3, 13 (26.0%) equal to 4 + 4, 20 (40.0%) equal to 4 + 5, 6 (12.0%) equal to 5 + 4, and in 3 cases (liver, lung, and a lymph node metastases) the Gleason score was not available (Table 5). Regarding the Grade Group, 8 tumors (16.0%) had a grade equal to 3, 13 (26.0%) had a grade score of 4, and 26 (52.0%) had a grade score equal to 5 (Table 5). Neoplastic cell enrichment ranged from 20% to 80% (mean, 37.8%). 

**Figure 7:**
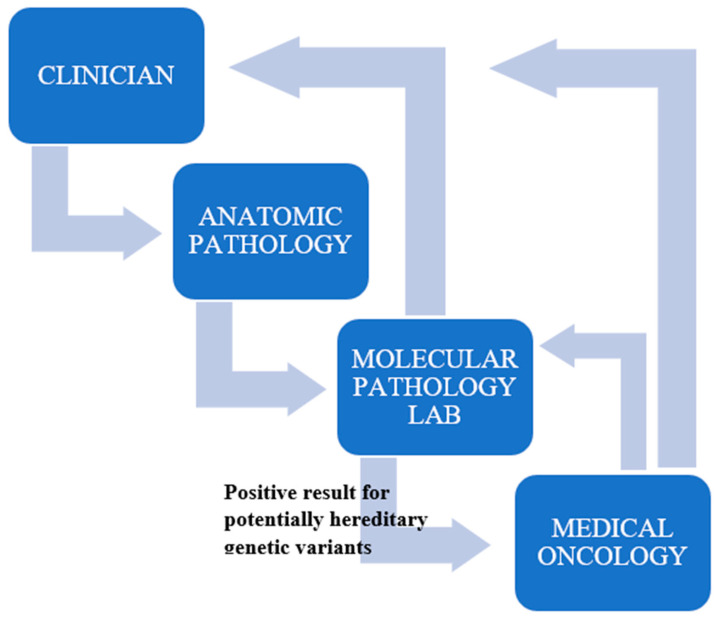
Scheme for management of patients with mCRPC.

All patients signed an informed consent to the genetic test performed on tumoral tissue and, in case the analysis revealed potentially hereditary genetic variants, to authorize to communicate data to the Cancer Genetics Clinic (IRCCS Policlinico di S. Orsola) and the latter to contact the patient to schedule a cancer genetic consultation (Figure 7).

### 4.2. NGS Analysis

DNA from the FFPE block was extracted using starting from two to four 10 μm FFPE sections under microscopic guidance according to the more representative area marked by a pathologist on a haematoxylin and eosin control-stained slide. Extracted DNA was quantified using a fluorometer (Qubit, Thermo Fisher Scientific, Waltham, MA, USA). 

The DNA was used for amplicon library preparation using a laboratory-developed multi-gene panel (customized Oncomine Tumor Specific Panel, Thermo Fisher Scientific). The panel allows amplifying a total of 1360 amplicons (88.73 kb, human reference sequence hg19/GRCh37) in the whole CDS (Coding Sequence) of the following 24 genes: *ARID1A*, *ATM*, *BAP1*, *BRIP1*, *BARD1*, *BRCA1*, *BRCA2*, *CDK12*, *CHEK1*, *CHEK2*, *FANCA*, *FANCL*, *IDH1*, *MLH1*, *MSH2*, *NBN*, *PALB2*, *MSH6*, *PMS2*, *PPP2R2A*, *RAD51B*, *RAD51C*, *RAD51D*, and *RAD54*. Briefly, about 30 ng of input DNA was used for NGS library preparation with the AmpliSeq Plus Library Kit 2.0 (Thermo Fisher Scientific, Waltham, MA, USA). Templates were then sequenced using an Ion 530 chip and the results were analyzed with the IonReporter tools (version 5.18, Thermo Fisher Scientific) and IGV software (Integrative Genome Viewer version 2.12.2—https://software.broadinstitute.org/software/igv/). According to the previously reported validation [41], only mutations present in at least 5% of the total number of reads analyzed and observed in both strands were considered for mutational calls. The Varsome tool (https://varsome.com/, accessed on March 2023) [42] was used to evaluate the ACMG classification, AMP score, and ClinVar classification of each mutation.

In the event of detection of pathogenic variants or VUS with allelic load ≥ 20% in the *BRCA1*/*BRCA2*, *MLH1*, *MSH2*, *MSH6*, *PMS2*, *RAD51*, *BAP1* genes or any other genes present in the panel to be reported ad hoc (potentially hereditary variants), the report of the Metropolitan Laboratory of Molecular Pathology is sent to the requesting clinician as well as to the Oncological Genetics Clinic for further case management.

## Figures and Tables

**Table 1:** Mutations detected in the analyzed cohort.

	Patients Harboring Pathogenic/VUS Variant(s) n = 50 (%)	Number of Pathogenic/VUS Variants n = 35 (%)	ACMG Classification	VAF Range
*BRCA1 ^£^*	3 (6.0)	3 (8.6)	3 Pathogenic	6–14%
*BRCA2 ^°^$%=^*	7 (14.0)	9 (25.7)	7 Pathogenic 2 VUS	6–44% 16–28%
*ARID1A*	1 (2.0)	1 (2.9)	1 VUS	51%
*ATM ^$=ç^*	6 (12.0)	6 (17.1)	1 Pathogenic 5 VUS	22% 14–51%
*BAP1 ^%^*	1 (2.0)	1 (5.7)	1 VUS	15%
*BARD1 ^$^*	1 (2.0)	1 (2.9)	1 VUS	27%
*BRIP1 ^£^*	1 (2.0)	1 (2.9)	1 Pathogenic	15%
*CHEK2 °*	1 (2.0)	1 (2.9)	1 VUS	11%
*CDK12 ^$^*	1 (2.0)	1 (2.9)	1 Pathogenic	14%
*FANCA ^&^*	2 (4.0)	2 (5.7)	2 Pathogenic	19–47%
*FANCL*	2 (4.0)	2 (5.7)	1 Pathogenic 1 VUS	26% 21%
*MLH1*	1 (2.0)	1 (2.9)	1 Pathogenic	53%
*PALB2*	1 (2.0)	1 (2.9)	1 Pathogenic	15%
*PIK3CA ^&^*	1 (2.0)	1 (2.9)	1 Pathogenic	48%
*PMS2*	2 (4.0)	2 (5.7)	2 VUS	12–53%
*PPP2R2A ^&^*	1 (2.0)	1 (2.9)	1 VUS	11%
*RAD51D ^ç^*	1 (2.0)	1 (2.9)	1 VUS	44%

VAF: Variant Allele Frequency; VUS: Variant of Uncertain Significance; *^£^* one case harbored concomitant *BRCA1* and *BRIP1* variants; ^ one case harbored concomitant *BRCA2* variants; ° one case harbored concomitant two *BRCA2* and one *CHEK2* variants; *^%^* one case harbored *BRCA2* and *BAP1* variants; ^=^ one case harbored *BRCA2* and *ATM* variants; *^$^* one case harbored concomitant *BRCA2*, *ATM*, *BARD1*, and *CDK12* mutations; *^&^* one case harbored concomitant *FANCA*, *PIK3CA*, and *PPP2R2A* variants; *^ç^* one case harbored concomitant *ATM* and *RAD51D* variants. Pathogenic classification includes both “pathogenic” and “likely pathogenic” variants.

**Table 2:** Number of mutated samples grouped according to the Gleason Score.

Gleason Score (n = 47) ^	*BRCA* Mut (%)	*BRCA* WT (%)	*p*-Value	HRR Mut (%)	HRR WT (%)	*p*-Value
4 + 3 (n = 8)	2 (25.0)	6 (75.0)	0.675	4 (50.0)	4 (50.0)	0.565
4 + 4 (n = 13)	3 (23.1)	10 (76.9)		6 (50.0)	7 (50.0)	
4 + 5 (n = 20)	3 (15.0)	17 (85.0)		7 (31.3)	13 (68.7)	
5 + 4 (n = 6)	0 (/)	6 (100)		4 (40.0)	2 (60.0)	

^ in three cases the Gleason Score was not available. *p*-Value: Chi-square Test.

**Table 3:** Number of mutated samples grouped according to Gleason Score. ^ in three cases Gleason Score was not available. *p*-Value: Chi-square Test.

Score Grade (n = 37) ^	BRCA Mut (%)	BRCA WT (%)	*p*-Value	HRR Mut (%)	HRR WT (%)	*p*-Value
3 (n = 8)	2 (33.3)	8 (66.7)	0.535	3 (50.0)	3 (50.0)	0.922
4 (n = 13)	3 (30.0)	13 (70.0)		5 (50.0)	5 (50.0)	
5 (n = 26)	3 (14.3)	26 (85.7)		8 (38.1)	13 (61.9)	

**Table 4:** Cases with the recommendation of confirming variants in germline DNA.

Case	Age	Mutated Gene	Somatic Mutation	VAF %	Confirmed in Germline DNA	ClinVar	ACMG
4	63	*BRCA2*	c.4131_4132insTGAGGA	44	YES	P	P
13	59	*MLH1*	c.794G>A	53	YES	Conflicting	LP
19	71	*BRCA2*	c.1546_1547del	24	In progress	NA	LP
27	73	*BRCA2* *BRCA2*	c.4913_4915delinsTTC c.4983T>G	28 21	In progress	NA P	VUS P
33	68	*PMS2*	c.1004A>G	53	In progress	VUS	VUS

VAF: Variant Allele Frequency; ACMG: American College of Medical Genetics classification; P: Pathogenic; LP: Likely Pathogenic; and NA: Not Available.

**Table 5:** Features of the analyzed cohort (mCRPC).

Samples Features	Values (%, n = 50)
**Analyzed specimens**	50
Surgical Resection	10 (7.5)
Biopsy	40 (92.5)
**Source of the analyzed specimens**	
Primary site (prostate)	43 (86.0)
Lymph node	3 (6.0)
Lung	2 (4.0)
Liver	1 (2.0)
Bladder	1 (2.0)
**Gleason Score**	
4 + 3	8 (16.0)
4 + 4	13 (26.0)
4 + 5	20 (40.0)
5 + 4	6 (12.0)
NA	3 (6.0)
**Grade Group**	
3	8 (16.0)
4	13 (26.0)
5	26 (52.0)
NA	3 (6.0)
**Neoplastic Cell Enrichment**	37.8% (20–80%)

NA: not available.

## Data Availability

All data is contained within the article.

## Supplementary Materials

The following supporting information can be downloaded at: https://www.mdpi.com/article/10.3390/ijms24108940/s1.

## References

[1] Sung H., Ferlay J., Siegel R.L., Laversanne M., Soerjomataram I., Jemal A., Bray F. (2021). Global Cancer Statistics 2020: GLOBOCAN Estimates of Incidence and Mortality Worldwide for 36 Cancers in 185 Countries. CA Cancer J. Clin..

[2] Giri V.N., Knudsen K.E., Kelly W.K., Cheng H.H., Cooney K.A., Cookson M.S., Dahut W., Weissman S., Soule H.R., Petrylak D.P. (2020). Implementation of Germline Testing for Prostate Cancer: Philadelphia Prostate Cancer Consensus Conference 2019. J. Clin. Oncol..

[3] Ewing C.M., Ray A.M., Lange E.M., Zuhlke K.A., Robbins C.M., Tembe W.D., Wiley K.E., Isaacs S.D., Johng D., Wang Y. (2012). Germline mutations in HOXB13 and prostate-cancer risk. N. Engl. J. Med..

[4] Kirby M., Hirst C., Crawford E.D. (2011). Characterising the castration-resistant prostate cancer population: A systematic review. Int. J. Clin. Pract..

[5] Scher H.I., Halabi S., Tannock I., Morris M., Sternberg C.N., Carducci M.A., Eisenberger M.A., Higano C., Bubley G.J., Dreicer R. (2008). Design and end points of clinical trials for patients with progressive prostate cancer and castrate levels of testosterone: Recommendations of the Prostate Cancer Clinical Trials Working Group. J. Clin. Oncol..

[6] Scott R.J., Mehta A., Macedo G.S., Borisov P.S., Kanesvaran R., El Metnawy W. (2021). Genetic testing for homologous recombination repair (HRR) in metastatic castration-resistant prostate cancer (mCRPC): Challenges and solutions. Oncotarget.

[7] Furlow B. NCCN: More Genetic Testing to Inform Prostate Cancer Management. Cancer Network. https://www.cancernetwork.com/view/nccn-more-genetic-testing-inform-prostate-cancer-management.

[8] Robinson D., Van Allen E.M., Wu Y.M., Schultz N., Lonigro R.J., Mosquera J.M., Montgomery B., Taplin M.E., Pritchard C.C., Attard G. (2015). Integrative Clinical Genomics of Advanced Prostate Cancer. Cell.

[9] Pritchard C.C., Mateo J., Walsh M.F., De Sarkar N., Abida W., Beltran H., Garofalo A., Gulati R., Carreira S., Eeles R. (2016). Inherited DNA-Repair Gene Mutations in Men with Metastatic Prostate Cancer. N. Engl. J. Med..

[10] Kote-Jarai Z., Leongamornlert D., Saunders E., Tymrakiewicz M., Castro E., Mahmud N., Guy M., Edwards S., O’Brien L., Sawyer E. (2011). BRCA2 is a moderate penetrance gene contributing to young-onset prostate cancer: Implications for genetic testing in prostate cancer patients. Br. J. Cancer.

[11] Carr T.H., Adelman C., Barnicle A., Kozarewa I., Luke S., Lai Z., Hollis S., Dougherty B., Harrington E.A., Kang J. (2021). Homologous Recombination Repair Gene Mutation Characterization by Liquid Biopsy: A Phase II Trial of Olaparib and Abiraterone in Metastatic Castrate-Resistant Prostate Cancer. Cancers.

[12] Robson M., Im S.A., Senkus E., Xu B., Domchek S.M., Masuda N., Delaloge S., Li W., Tung N., Armstrong A. (2017). Olaparib for Metastatic Breast Cancer in Patients with a Germline BRCA Mutation. N. Engl. J. Med..

[13] Golan T., Hammel P., Reni M., Van Cutsem E., Macarulla T., Hall M.J., Park J.O., Hochhauser D., Arnold D., Oh D.Y. (2019). Maintenance Olaparib for Germline BRCA-Mutated Metastatic Pancreatic Cancer. N. Engl. J. Med..

[14] Moore K., Colombo N., Scambia G., Kim B.G., Oaknin A., Friedlander M., Lisyanskaya A., Floquet A., Leary A., Sonke G.S. (2018). Maintenance Olaparib in Patients with Newly Diagnosed Advanced Ovarian Cancer. N. Engl. J. Med..

[15] Kaufman B., Shapira-Frommer R., Schmutzler R.K., Audeh M.W., Friedlander M., Balmana J., Mitchell G., Fried G., Stemmer S.M., Hubert A. (2015). Olaparib monotherapy in patients with advanced cancer and a germline BRCA1/2 mutation. J. Clin. Oncol..

[16] De Bono J., Mateo J., Fizazi K., Saad F., Shore N., Sandhu S., Chi K.N., Sartor O., Agarwal N., Olmos D. (2020). Olaparib for Metastatic Castration-Resistant Prostate Cancer. N. Engl. J. Med..

[17] Hussain M., Mateo J., Fizazi K., Saad F., Shore N., Sandhu S., Chi K.N., Sartor O., Agarwal N., Olmos D. (2020). Survival with Olaparib in Metastatic Castration-Resistant Prostate Cancer. N. Engl. J. Med..

[18] O’Connor M.J. (2015). Targeting the DNA Damage Response in Cancer. Mol. Cell.

[19] Farmer H., McCabe N., Lord C.J., Tutt A.N., Johnson D.A., Richardson T.B., Santarosa M., Dillon K.J., Hickson I., Knights C. (2005). Targeting the DNA repair defect in BRCA mutant cells as a therapeutic strategy. Nature.

[20] Pommier Y., O’Connor M.J., de Bono J. (2016). Laying a trap to kill cancer cells: PARP inhibitors and their mechanisms of action. Sci. Transl. Med..

[21] USPI (2020). Rucaparib Tablets, for Oral Use. https://www.ema.europa.eu/en/documents/pip-decision/p/0242/2020-ema-decision-22-june-2020-granting-product-specific-waiver-rucaparib-camsylate-emea-002760_en.pdf.

[22] Prostate Cancer NCCN Guidelines Version 2. https://www.nccn.org/professionals/physician_gls/default.aspx.

[23] Abida W., Patnaik A., Campbell D., Shapiro J., Bryce A.H., McDermott R., Sautois B., Vogelzang N.J., Bambury R.M., Voog E. (2020). Rucaparib in Men With Metastatic Castration-Resistant Prostate Cancer Harboring a BRCA1 or BRCA2 Gene Alteration. J. Clin. Oncol..

[24] Smith M.R., Scher H.I., Sandhu S., Efstathiou E., Lara P.N., Yu E.Y., George D.J., Chi K.N., Saad F., Stahl O. (2022). Niraparib in patients with metastatic castration-resistant prostate cancer and DNA repair gene defects (GALAHAD): A multicentre, open-label, phase 2 trial. Lancet Oncol..

[25] Damodaran S., Berger M.F., Roychowdhury S. (2015). Clinical tumor sequencing: Opportunities and challenges for precision cancer medicine. Am. Soc. Clin. Oncol. Educ. Book.

[26] Capoluongo E., Ellison G., Lopez-Guerrero J.A., Penault-Llorca F., Ligtenberg M.J.L., Banerjee S., Singer C., Friedman E., Markiefka B., Schirmacher P. (2017). Guidance Statement On BRCA1/2 Tumor Testing in Ovarian Cancer Patients. Semin. Oncol..

[27] Bass B.P., Engel K.B., Greytak S.R., Moore H.M. (2014). A review of preanalytical factors affecting molecular, protein, and morphological analysis of formalin-fixed, paraffin-embedded (FFPE) tissue: How well do you know your FFPE specimen?. Arch. Pathol. Lab. Med..

[28] Compton C.C., Robb J.A., Anderson M.W., Berry A.B., Birdsong G.G., Bloom K.J., Branton P.A., Crothers J.W., Cushman-Vokoun A.M., Hicks D.G. (2019). Preanalytics and Precision Pathology: Pathology Practices to Ensure Molecular Integrity of Cancer Patient Biospecimens for Precision Medicine. Arch. Pathol. Lab. Med..

[29] Einaga N., Yoshida A., Noda H., Suemitsu M., Nakayama Y., Sakurada A., Kawaji Y., Yamaguchi H., Sasaki Y., Tokino T. (2017). Assessment of the quality of DNA from various formalin-fixed paraffin-embedded (FFPE) tissues and the use of this DNA for next-generation sequencing (NGS) with no artifactual mutation. PLoS ONE.

[30] The Cancer Genome Atlas Research Network (2015). The Molecular Taxonomy of Primary Prostate Cancer. Cell.

[31] de Bono J.S., Fizazi K., Saad F., Shore N., Sandhu S.K., Mehra N., Kolinsky M., Roubaud G., Özgüroǧlu M., Matsubara N. (2019). 847PD—Central, prospective detection of homologous recombination repair gene mutations (HRRm) in tumour tissue from >4000 men with metastatic castration-resistant prostate cancer (mCRPC) screened for the PROfound study. Ann. Oncol..

[32] Castro E., Goh C., Olmos D., Saunders E., Leongamornlert D., Tymrakiewicz M., Mahmud N., Dadaev T., Govindasami K., Guy M. (2013). Germline BRCA mutations are associated with higher risk of nodal involvement, distant metastasis, and poor survival outcomes in prostate cancer. J. Clin. Oncol..

[33] Castro E., Romero-Laorden N., Del Pozo A., Lozano R., Medina A., Puente J., Piulats J.M., Lorente D., Saez M.I., Morales-Barrera R. (2019). PROREPAIR-B: A Prospective Cohort Study of the Impact of Germline DNA Repair Mutations on the Outcomes of Patients With Metastatic Castration-Resistant Prostate Cancer. J. Clin. Oncol..

[34] Carter H.B., Helfand B., Mamawala M., Wu Y., Landis P., Yu H., Wiley K., Na R., Shi Z., Petkewicz J. (2019). Germline Mutations in ATM and BRCA1/2 Are Associated with Grade Reclassification in Men on Active Surveillance for Prostate Cancer. Eur. Urol..

[35] Castro E., Goh C., Leongamornlert D., Saunders E., Tymrakiewicz M., Dadaev T., Govindasami K., Guy M., Ellis S., Frost D. (2015). Effect of BRCA Mutations on Metastatic Relapse and Cause-specific Survival After Radical Treatment for Localised Prostate Cancer. Eur. Urol..

[36] Schweizer M.T., Sivakumar S., Tukachinsky H., Coleman I., De Sarkar N., Yu E.Y., Konnick E.Q., Nelson P.S., Pritchard C.C., Montgomery B. (2021). Concordance of DNA Repair Gene Mutations in Paired Primary Prostate Cancer Samples and Metastatic Tissue or Cell-Free DNA. JAMA Oncol..

[37] Russo A., Incorvaia L., Capoluongo E., Tagliaferri P., Gori S., Cortesi L., Genuardi M., Turchetti D., De Giorgi U., Di Maio M. (2022). Implementation of preventive and predictive BRCA testing in patients with breast, ovarian, pancreatic, and prostate cancer: A position paper of Italian Scientific Societies. ESMO Open.

[38] Mateo J., Carreira S., Sandhu S., Miranda S., Mossop H., Perez-Lopez R., Nava Rodrigues D., Robinson D., Omlin A., Tunariu N. (2015). DNA-Repair Defects and Olaparib in Metastatic Prostate Cancer. N. Engl. J. Med..

[39] Crocetto F., Russo G., Di Zazzo E., Pisapia P., Mirto B.F., Palmieri A., Pepe F., Bellevicine C., Russo A., La Civita E. (2022). Liquid Biopsy in Prostate Cancer Management-Current Challenges and Future Perspectives. Cancers.

[40] McFarland T.R., Thomas V.M., Nussenzveig R., Gebrael G., Sayegh N., Tripathi N., Sahu K.K., Goel D., Maughan B.L., Sirohi D. (2022). Detection of BRCA1, and BRCA2 Alterations in Matched Tumor Tissue and Circulating Cell-Free DNA in Patients with Prostate Cancer in a Real-World Setting. Biomedicines.

[41] de Biase D., Acquaviva G., Visani M., Sanza V., Argento C.M., De Leo A., Maloberti T., Pession A., Tallini G. (2020). Molecular Diagnostic of Solid Tumor Using a Next Generation Sequencing Custom-Designed Multi-Gene Panel. Diagnostics.

[42] Kopanos C., Tsiolkas V., Kouris A., Chapple C.E., Albarca Aguilera M., Meyer R., Massouras A. (2019). VarSome: The human genomic variant search engine. Bioinformatics.

