# Supplementary material for: Multi-Gene Next-Generation Sequencing Panel for Analysis of BRCA1/BRCA2 and Homologous Recombination Repair Genes Alterations Metastatic Castration-Resistant Prostate Cancer
Source: Int J Mol Sci. 2023 May 18;24(10):8940. doi: 10.3390/ijms24108940 (PMC10219522; doi:10.3390/ijms24108940)
Supplement: Supplementary file 1 [file ijms-24-08940-s001.zip › Supplementary Figure S1.pdf]

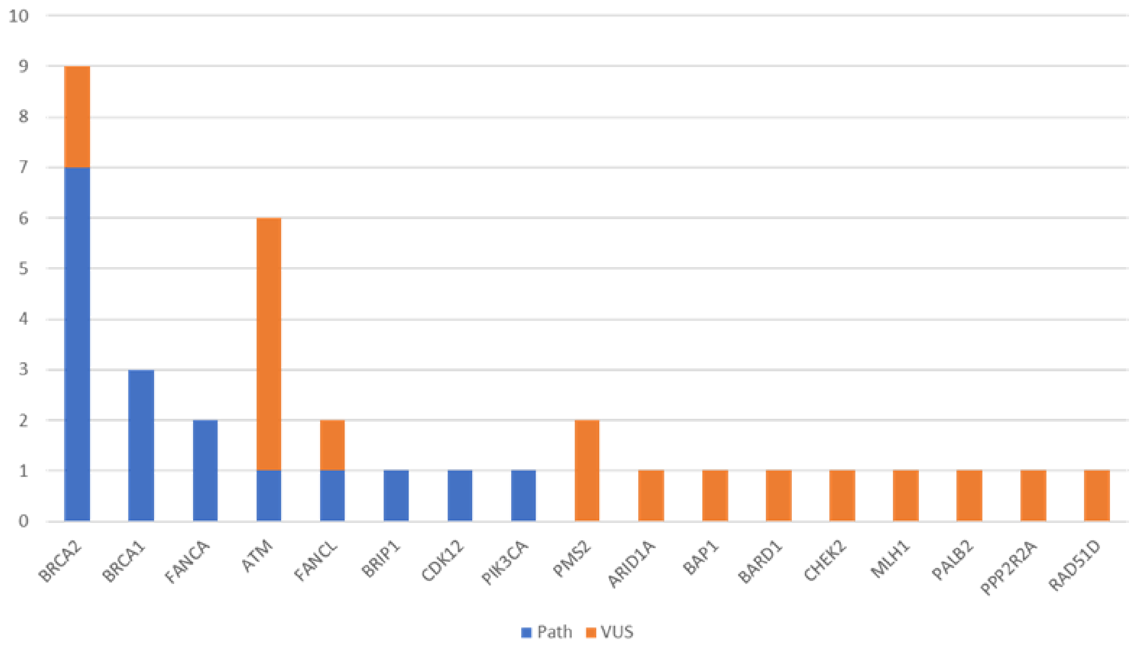

Figure S1: Total number of mutations in the analyzed cohort. Path: Pathogenic and Likely Pathogenic mutations. VUS: variant of uncertain significance. Y axis: number of samples
